# Building and Advancing Coalition Capacity to Promote Health Equity: Insights from the Health Equity Collective's Approach to Addressing Social Determinants of Health

**DOI:** 10.1089/heq.2021.0012

**Published:** 2021-12-27

**Authors:** Jemima C. John, Tanweer Kaleemullah, Heidi McPherson, Kallol Mahata, Robert B. Morrow, Deborah Bujnowski, Alicia Johnston, Melisa Danho, Nadia Siddiqui, Michael T. Walsh, Sean A. Haley, Anwar M. Sirajuddin, Timothy Schauer, Mon-Ju Wu, Ruth Rechis, Esperanza Galvan, Nancy Correa, Nikki Browning, Deborah Ganelin, Jennifer Gonzalez, Staci Lofton, Deborah Banerjee, Shreela V. Sharma

**Affiliations:** ^1^ Michael and Susan Dell Center for Healthy Living, The University of Texas Health Science Center at Houston School of Public Health, Houston, Texas, USA.; ^2^ Harris County Public Health, Houston, Texas, USA.; ^3^ American Heart Association, Houston, Texas, USA.; ^4^ Patient Care Intervention Center, Houston, Texas, USA.; ^5^ Humana, Inc., Houston, Texas, USA.; ^6^ Texas Health Institute, Austin, Texas, USA.; ^7^ The University of Texas MD Anderson Cancer Center, Houston, Texas, USA.; ^8^ Center for Civic and Public Policy Improvement, Houston, Texas, USA.; ^9^ Cornerstone, Houston, Texas, USA.; ^10^ Department of Psychiatry and Behavioral Sciences, The University of Texas Health Science Center at Houston, Houston, Texas, USA.; ^11^ Texas Children's Hospital, Houston, Texas, USA.; ^12^ Houston Food Bank, Houston, Texas, USA.; ^13^ Memorial Hermann Health System, Houston, Texas, USA.; ^14^ City of Houston Department of Health and Human Services, Houston, Texas, USA.

**Keywords:** Coalition, collective impact, population health, Community Information Exchange

## Abstract

This article presents the structure and function of the Health Equity Collective in developing a systemic approach to promoting health equity across the Greater Houston area. Grounded in Kania and Kramer's five phases of collective impact for coalition building, The Collective operationalizes its mission through its backbone team, steering committees, and eight workgroups; each has goals that mutually reinforce and advance its vision. To date, Phase I (generating ideas), Phase II (initiating action), and Phase III (organizing for impact) have been completed. Phases IV (implementation) and Phase V (sustainability) are currently underway.

## Background

The United States spends far more per capita than most developed nations, yet still falls behind on quality of life (QOL) and health outcomes.^1,2^ Financial investments in services that address social determinants of health (SDoH) indicators would significantly improve these health outcomes.^3,4^ These indicators contribute to 80% of individual health outcomes.^5–7^ Poor and uneven investments in these resources have resulted in health inequities across communities of color and low-income populations.^8–11^ Moreover, the severe acute respiratory syndrome coronavirus (SARS-CoV-2) pandemic has worsened, and social vulnerabilities in these communities.^12,13^ Thus, timely addressing SDOH needs can improve population health and health equity.^5,6^

Harris County, which contains Houston and other smaller cities, has over 4.7 million residents and is the third-most populous county in the United States.^14^ Across the county, life expectancy can vary by 24 years based on residential zip codes; 50% of adults are considered obese, and 20% are uninsured.^15^ In response, the Harris County Public Health's *Harris Cares* report provided a visionary outlook for improving the county's health; recommendations included needs to improve systems interoperability and a focus on upstream prevention and effective integration of Texas's health and social services.^15^ Such actions necessitate multisector alignment and collective impact approaches.^16,17^

Thus, the Health Equity Collective (the Collective) was established in December 2018 with a priority goal of food insecurity reduction by 5% in 2025 and a mission to establish a sustainable, data-driven, human-centered ecosystem of care that targets SDoH needs across Greater Houston. This article presents the Collective's structure and progress in this space.^17^

## Establishing a Collective Impact Structure

According to Schuler and Koka, coalitions must first address “problem ambiguity, user dimensionality, structural barriers, and a mindset of competition among social service organizations” as common barriers to effectively tackle societal issues.^18^ To circumvent these potential challenges, the Collective adopted Kania and Kramer's conditions of common agenda, shared measurement systems, mutually reinforcing activities, continuous communication, and backbone support, which are currently cemented across five phases: (I) generating ideas and host dialogues, (II) initiating action, (III) organizing for impact, (IV) beginning implementation, and (V) sustaining action and impact.^16,17^

## Capacity Building and Phases of Action

In Phase I, coleads from the University of Texas Health Science Center at Houston, School of Public Health (UTSPH), American Heart Association, and Harris County Public Health conducted over 200 one-on-one listening meetings with prospective Collective members and community stakeholders from December 2018 to December 2019. An SDoH screening and data-sharing landscape scan between Greater Houston health care and community-based organizations (CBOs) were also produced to better understand and establish possible data-sharing relationships that would benefit the community.

In August 2019, the Collective also convened 85+ key stakeholders to help shape its focus. Deliberations revealed care coordination and data-sharing capacity across the health and social services sectors as the approaches best suited to address SDoH needs across the Greater Houston. During this period, our mission to achieve health equity was also cemented with unanimous consent. A second convening in February 2020 with executive leadership from 25 Greater Houston organizations resulted in recommendations for an information exchange ecosystem starting first with the development of a Community Information Exchange (CIE), which would allow for referral and care coordination between social service providers in the Greater Houston area.

Backbone coleads also reached out to local and national philanthropic entities to develop a sustainable funding strategy for the Coalition. Activities conducted in Phase I not only solidified consensus on the Coalition's mission, goals, structure, and strategic direction, but also laid the groundwork for Phases II and III of collective impact action.

**FIG. 1.**
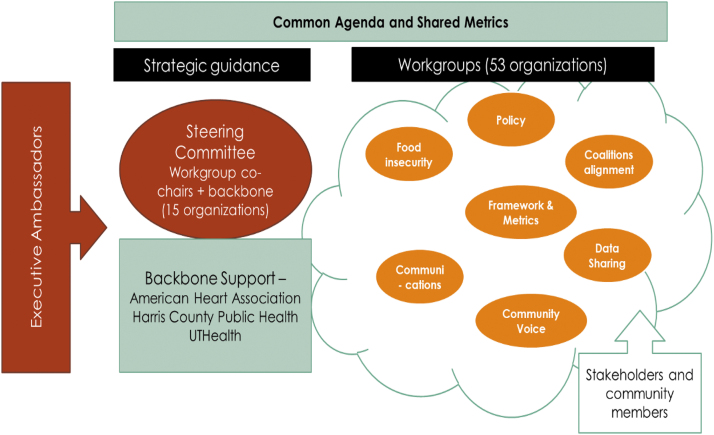
The Health Equity Collective's organizational structure.

**FIG. 2.**
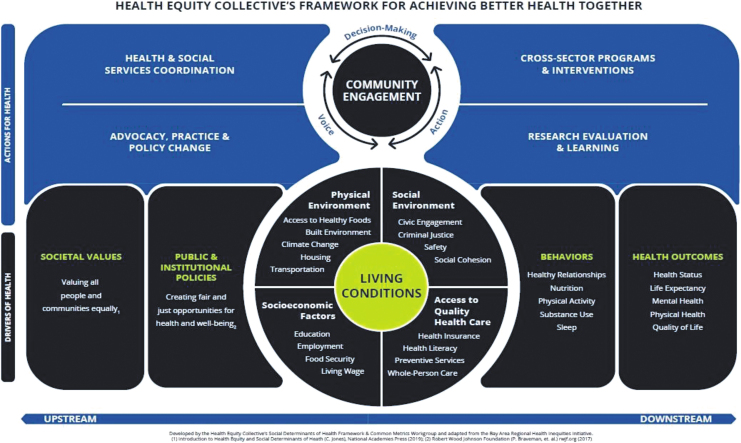
The Health Equity Collective framework for achieving health equity.

Central to Phases II and III was the formation of the Collective's structure and functions (Table 1 and Fig. 1). The three coleading backbone organizations also drafted a memorandum of understanding to articulate the Collective's roles and responsibilities. Presently, 18 organizations form the Collective's steering committee and over 53 organizations actively participate in its workgroups. Concurrently, the Collective's logic framework for advancing health equity was developed and adapted from the Bay Area Regional Health Inequities Initiative (BARHII) Framework for Reducing Health Inequities (Fig. 2).^19^

**Table 1.** The Health Equity Collective Structure of Background Support, Executive Ambassadors, and Workgroups

Backbone Leads Three-organization backbone support for the Greater Houston Coalition for SDoH: American Heart Association, Harris County Public Health, and UTHealth School of Public Health		
Executive Ambassadors Engage community and business leaders to foster policy, systems, and environmental changes that will lead to improvements in health outcomes		

Workgroups	Workgroup descriptions	Core goals of each workgroup
Coalition Alignment	Connect the efforts of the Health Equity Collective to the many other coalitions and collective efforts addressing components of SDoH across the region.	(1) Identify coalitions in the Greater Houston that are addressing SDoH. (2) Collect information on the goals and activities of each coalition.
Community Voice	Our collective effort must be rooted in meeting the SDoH needs across our community—as informed by those who are impacted by inequities.	(1) Conduct a scan of grassroots community efforts across the Coalition. (2) Agree on approach for informing GHC-SDoH efforts with community voice.
Communications	Develop strategies for increasing awareness of the GHC on SDoH impact and promoting engagement in SDoH efforts across our region.	(1) Brand and digital presence. (2) Establish initial marketing plan.
Data-sharing Ecosystem	Focuses on establishing data ecosystem, network for data-sharing, and technical capacity for population-level data analysis.	(1) Recommend options for ecosystem interoperability, building upon existing data-sharing efforts with focus on HIE connection to CIE or similar function. (2) Contribute to the development of shared RFP language for interoperability. (3) Recommend screening, care coordination, data collection and data flows. (4) Contribute data sharing efforts to Coalition's comprehensive landscape.
Food Security	Develop a shared set of evidenced-based interventions to improve food security and healthy food intake across the Greater Houston region. This will build on existing food security efforts.	(1) Conduct a landscape scan of food security interventions. (2) Compile existing learnings, best practices, and recommendations. (3) Contribute food security efforts to the Coalition's comprehensive landscape. (4) Link Houston Food Systems Collaborative and determine shared strategies. (5) Recommend food interventions for broader implementation. (6) Develop language and framework for closed-loop referral process.
SDoH Policy	Connect the Coalition's SDoH efforts to existing policy platforms and SDoH policy efforts that could impact the Greater Houston area.	(1) Assess and report on the current SDoH policy opportunities for inclusion on the Coalition's comprehensive landscape. (2) Recommend an initial set of SDoH policies to support the work of the Coalition.
SDoH Framework and Common Metrics	Review existing SDoH frameworks to select and establish a framework to benchmark the broad efforts of this collective impact across the Greater Houston region.	(1) Identify framework(s) to utilize in developing a broader SDoH approach for the Coalition's work and recommend a plan for its application to the GHC SDoH work. (2) Determine metrics of success to be used in proof-of-concept for outcomes.

Each workgroup is supported by one member of the backbone organization.

CIE, Community Information Exchange; RFP, request for proposal; SDoH, social determinants of health.

During Phases I and II, membership grew from 100 members and 75 organizations to over 375 members representing 130 organizations sectors across the Greater Houston area.

The Collective is now in its Phase IV (2021–2022) of the collective impact approach, and has initiated the development of cloud-based CIE. Using a federated model approach, the CIE will allow multiple organizations with varied technologies to connect, coordinate care, and serve their populations' social needs. Once established, the CIE will connect with the health care sector and will allow for bidirectional referrals and care coordination with health care and behavioral health entities. The CIE architecture will include the following:

(1) Developing and implementing a resource data infrastructure to convene disparate sources of data to address SDoH needs.
(2) Developing and implementing the referral network infrastructure to support referrals between social service organizations and to track the impact of referrals on clients' social needs and behavioral outcomes.
(3) Developing a vendor agnostic infrastructure to link the CIE and health care organizations to facilitate care coordination. The Collective workgroups and its steering committee will work in tandem to support CIE efforts. As immediate next steps, the Collective will also initiate a governance process for the CIE as well as facilitate its 12-month proof-of-concept. As we enter Phase V of collective impact in 2021, the principal focus will be to launch the CIE proof-of-concept and related governance elements.

## Discussion

Addressing key SDoH indicators is rightfully a global health priority^20^ and a recognized focus for many health, community, institutional, and business entities across the Greater Houston area.^15^ Achieving health equity demands an accelerated pivot toward improved systems interoperability and a clear focus on upstream prevention and integration of health and social services. These transformational shifts have been operationalized by the newly formed Health Equity Collective—a coalition that has convened cross-sector organizational support to address and promote population well-being.^21,22^

Formed in 2018, the Collective has prioritized the development and implementation of a sustainable, data-driven ecosystem of care to achieve health equity. This article adds to the current body of literature on SDoH by describing the structure and functions of this coalition's work across the Greater Houston area and details our collective impact-driven framework to address our mission and future work in this area.

The collective impact approach to coalition-building posits that organizations from diverse sectors must work together around a shared vision to make lasting social change. Working toward this shared vision is greatly enhanced because of common measurement approaches, activities that reinforce the Collective's agenda, ongoing communication, and the continuous support of a backbone team, keeping the participating institutions grounded in a shared vision.^16,17^ The success of the collective impact approach has been mirrored in other existing U.S. coalitions; their successes have informed the Collective's work and our roadmap for looking ahead.

An example of a multipronged collective impact initiative is “Memphis Fast Forward,” which was established in 2008 to launch “Operation: Safe Community.”^23,24^

The coalition made significant headway on its prosecution and policing metrics, but struggled to achieve progress on the third metric: violence prevention. To recalibrate, coalition members worked in unison to identify and apply for funding mechanisms and were successfully awarded a grant by the U.S. Department of Justice.^23,24^ Similarly, our coalition has learned to adapt and integrate an iterative process of learning and action, which has collectively moved us closer to satisfying goals that meet our shared agenda.

Another collective impact initiative is the “Communities that Care” Coalition, launched in 2003 by Franklin County in Massachusetts to address teen substance abuse. The shared vision was to improve parents' behaviors and attitudes on teen substance abuse through parental training and “parents teach other parents” sessions. To inform coalition efforts, members engaged in various data collection and sharing activities (focus groups, surveys, etc.) that summarily found family dinner messaging to be the best approach for achieving the coalition's goal.^23,25^

Armed with this information, the Coalition retooled its messaging strategies to improve outreach efforts.^23,25^ Efforts not only demonstrated a willingness to be quick-thinking and flexible but also kept the coalition on track with its overall goal, which remained unchanged.

Indeed, insights from these coalitions have provided directions for navigating challenges—many of which we have since turned into opportunities for growth and innovation. Through these and our own experiences, we found that convening institutions—that had their own priorities and way of doing things—around a shared vision to be occasionally difficult, yet manageable. For example, grant proposals and technology investments that were pursued as a coalition had better funding and sustainability prospects versus organizations that acted independently and within silos. Ongoing participation across backbone, steering committee, workgroup, and quarterly sessions greatly reinforced the importance of interoperability and “buy in” to the Collective’ shared agenda.

Second, an inherent challenge to building and maintaining a healthy coalition is the demand for committed leadership, effort beyond primary job responsibilities, and time investment. We experienced these “organizational capacity challenges” as a coalition. However, in our case, we found that an evolving and growing coalition of new members at the executive level or within workgroups brought a much-needed infusion of support, enthusiasm, expertise, and new ideas that pushed the vision of the Collective forward.

In particular, the work by the Food Security workgroup and Framework and Common Metrics workgroup members has proven transformational in advancing the Collective's health equity vision. As examples, the Food Security workgroup's conduct of (1) a landscape scan of food security and healthy food intake interventions/programs and (2) a comprehensive qualitative assessment of organizations' capacity to address food security (especially during the COVID-19 pandemic) will inform best practices and understandings for how organizations and the Collective can better support food security efforts across the county.

Similarly, the Frameworks and Common Metrics workgroup has worked diligently to develop a framework that identifies four main disciplines of health action that will influence identified “drivers of health” in the county. This model serves as a railroad for how the Collective will operationalize its vision and is quintessential for keeping the Collective grounded in its mission. These successful workgroup efforts and ongoing equally vital work by other workgroups have been supported by continuous communication, support, and iterative feedback from backbone coleads, steering committee leadership, and our executive ambassadorship team.

Lastly, through listening sessions with CBOs, qualitative assessments (Food Security workgroup), and ongoing networking and strategy sessions, we also realized that improvements in interoperability in screening protocols as in technology platforms are equally critical. Like other coalitions,^26^ the Health Equity Collective is uniquely positioned to leverage its partnerships to create standardized SDoH screening tools for various needs (food insecurity, housing insecurity, etc.) to facilitate both screening efficiency and care coordination efforts across health and social care sectors.

In conclusion, as the Collective expands, the work of diverse-sector coalition members will continue to influence how we implement an integrative framework for SDoH in the Greater Houston area. Looking ahead, this work could prove transformational in serving as a blueprint for how diverse sectors can work together to leverage their health and social services in tackling upstream QOL predictors.

## Notes

**Cite this article as:** John JC, Kaleemullah T, McPherson H, Mahata K, Morrow RB, Bujnowski D, Johnston A, Danho M, Siddiqui N, Walsh Jr MT, Haley SA, Sirajuddin AM, Schauer T, Wu M-J, Rechis R, Galvan E, Correa N, Browning N, Ganelin D, Gonzalez J, Lofton S, Banerjee D, Sharma SV (2021) Building and advancing coalition capacity to promote health equity: insights from the Health Equity Collective's approach to addressing social determinants of health, *Health Equity* 5:1, 872–878, DOI: 10.1089/heq.2021.0012.

## Abbreviations Used

CBO: community-based organization
CIE: Community Information Exchange
QOL: quality of life
SDoH: social determinants of health
